# Prevalence of Overweight and Obese Prepregnancy BMI and Excessive Gestational Weight Gain Using Asian-Specific Cutoffs Among Asian and Mixed-Asian Women Living in Hawaii: A Retrospective Cohort Study

**DOI:** 10.1007/s10995-022-03560-w

**Published:** 2022-10-10

**Authors:** Y. Daida, K. Pedula

**Affiliations:** Center for Integrated Healthcare Research, Kaiser Permanente Hawai’i, 501 Alakawa St Suite 201, Honolulu, HI 96817 USA

**Keywords:** Asian, Mixed-race, Body Mass Index, Reclassification, Gestational weight gain

## Abstract

**Introduction:**

The use of Asian-specific Body Mass Index (aBMI) cutoffs may be more appropriate than general World Health Organization BMI (gBMI) cutoffs in determining recommended gestational weight gain (GWG) for Asian women. Since aBMI cutoffs are lower than gBMI, more Asian women will be reclassified into higher aBMI categories from gBMI. The prevalence of reclassification and its impact on GWG are not known.

**Methods:**

We utilized the electronic health records of 8886 Kaiser Permanente Hawaii members aged ≥ 18 with a singleton live birth. Prepregnancy BMI was first classified using gBMI criteria, then aBMI criteria. BMI categories were “underweight”, “normal”, “overweight” and “obese”; GWG was classified into lower (“lGWG”), met (“mGWG”), and exceed (“eGWG”) GWG per WHO recommendations. Self-reported race/ethnicity include Asian, Asian + Pacific Islander, and Asian + white. Multiple logistic regression was used to estimate adjusted odds of reclassification. The Cochran-Mantel–Haenszel test was used to evaluate associations between race/ethnicity and GWG.

**Results:**

> 40% of women in each racial/ethnic group were reclassified. Asian + Pacific Islander women had significantly higher odds of being reclassified (p < .0001). In the normal gBMI and aBMI category, Asian + Pacific Islander women had the largest eGWG group. In the overweight gBMI category, Asian + Pacific Islander women had the largest eGWG group; in the overweight aBMI category, Asian + white women had the largest eGWG group.

**Discussion:**

A sizable percent of women were reclassified into higher BMI categories when aBMI was applied. Mixed-race Asian women were more likely to exceed GWG recommendations than Asian women.

## Significance Statement

*What is already known on this subject?* Asian women are at higher risk for obesity-related disease than white women with the same BMI. Studies suggest that aBMI should be used to estimate optimal GWG goals for Asian women.

*What does this study add?* In changing from gBMI to aBMI, at least 40% of Asian and mixed-race Asian women were reclassified into higher BMI categories. Asian + Pacific Islander women were more likely to be reclassified to a higher BMI category when aBMI was applied. More mixed-race Asian women exceeded recommended GWG than Asian women.

## Introduction

Excessive gestational weight gain (GWG) increases the burden of poor maternal and infant outcomes such as gestational diabetes (GDM), preeclampsia, and macrosomia. Each year, 6% to 9% of women develop GDM and the percentage of women with GDM increased 56% between 2000 and 2010 (Centers for Disease Control & Prevention, 2018). Preeclampsia occurs in about 4% of pregnant women in the United States (American College of Obstetricians and Gynecologists Task Force on Hypertension in Pregnancy, 2013). The Institute of Medicine (IOM) has published GWG recommendations to optimize health outcomes for both the woman giving birth and her infant (Institute of Medicine & and National Research Council Committee to Reexamine IOM Pregnancy Weight Guidelines, 2009). GWG is based on physiologic and metabolic changes that take place during pregnancy. Beyond weight gain due to placenta, fetus, amniotic fluid and hypertrophy of maternal tissues, additional gains in weight are due to increase in fat mass (Rasmussen et al. 2009). Clinicians in the U.S. have adopted the IOM’s recommendations and use them to provide weight gain goals to their patients. However, 48% of women in the U.S. exceed these recommendations (Branum, 2016). While poor maternal and child outcomes affect all racial/ethnic groups, Asian women are at higher risk for developing GDM (Shah et al., 2021) and suffer complications from preeclampsia (Minhas et al., 2021).

IOM recommends that during pregnancy, underweight women gain 28–40 pounds, normal weight women gain 25–35 pounds, overweight women gain 15–25 pounds, and obese women gain 11–20 pounds. IOM’s GWG recommendations (Institute of Medicine, 2009) are based on a woman’s prepregnancy body mass index (BMI, calculated as kg/m^2^) as defined by World Health Organization (WHO) for the general population. General BMI (gBMI) cutoffs are < 18.5 kg/m^2^, underweight; 18.5–24.9 kg/m^2^, normal weight; 25–29.9 kg/m^2^, overweight; and > 30 kg/m^2^, obese. However, WHO also has Asian-specific BMI (aBMI) cutoffs, which are lower than gBMI: 18.5–22.9 kg/m^2^, normal weight; 23–24.9 kg/m^2^, overweight; ≥ 25 kg/m^2^, obese (World Health Organization and Regional Office for the Western Pacific, 2000).

Previous research shows that Asian individuals with the same BMI as white individuals have higher obesity-related disease risks (Deurenberg-Yap et al., 2002; Lin et al., 2002; World Health Organization, 2004). Underlying this is the fact that body fat percent and distribution differ: Asian individuals have a higher percentage of body fat (Deurenberg et al., 2002) and upper-body fat (Lim et al., 2011), both of which are pro-inflammatory. BMI is widely used as an estimate of body fat (Freedman et al., 2013; Garrow & Webster, 1985; Wohlfahrt-Veje et al., 2014), and GWG guidelines reflect the importance of maternal prepregnancy body fat (as estimated by BMI) for outcome of pregnancy (Institute of Medicine, 2009; Institute of Medicine & and National Research Council Committee to Reexamine IOM Pregnancy Weight Guidelines, 2009). Since aBMI is a better reflection of body fat in Asian women, using gBMI to estimate GWG in Asian populations may unintentionally lead to excessive weight gain. The appropriateness of using using gBMI to estimate GWG goals in Asian populations is debatable, and studies have been published in Japan (Morisaki et al., 2017), Korea (Choi et al., 2017), China (Wu et al., 2018), Vietnam (Ota et al., 2011), Singapore (Ee et al., 2014) using aBMI instead of gBMI to assess optimal weight gain.

Recent evidence suggests that Asian women experience higher rates of GDM at lower gBMI levels (Bryant et al., 2014; Oteng-Ntim et al., 2013; Wong, 2012). Shah et al. (2011) found that using gBMI as a screening tool for GDM in Asian women identified only 25% of the study population, compared with > 76% of Black, 58% of Latina, and 46% of Caucasian women. One study found that among individuals with GDM, South Asian women had higher skinfold thickness and serum leptin (associated with insulin resistance and diabetes) even though their gBMI was lower than that of white women (Sommer et al., 2015). Furthermore, Yang et al. (2021) found that risks for preeclampsia increased more rapidly with gBMI for Chinese women than Swedish women.

The Asian population in the U.S. grew 81% from 2000 to 2019, making it the fastest growing growing racial or ethnic group. Furthermore, this population is increasingly diversifying, as reflected by the number of births of multiracial infants. For example, in 2013, 24% of infants were Asian + white and 2% were Asian + Pacific Islander (Pew Research Center, 2015). To date, only one study has compared prepregnancy BMI classifications using both gBMI and aBMI (Gao et al., 2020), and it reported on single-race Asian groups only. Moreover, no studies have reported changes in prevalence of excessive GWG using both gBMI and aBMI.

The goal of this study was to determine the prevalence of prepregnancy BMI reclassification in Asian and mixed-race Asian women when using aBMI. We also investigated which groups of women were most likely to be reclassified, and describe the prevalence of excessive weight gain in this paper.

## Methods

This retrospective population-based cohort study utilized the electronic health records (EHRs) of 8886 Kaiser Permanente Hawaii members who were at least 18 years old and gave birth to live singletons at ≥ 37 weeks (calculated from date of last menstrual period) between January 1, 2006, and December 31, 2019. Women with missing data on age, prepregnancy BMI, GWG, and race/ethnicity were excluded. Hispanic and Black women were also excluded. Each eligible patient was included once in the sample; if a woman had multiple pregnancies during the study period, only the most recent pregnancy was included for analyses. The following measures were available in the EHR: maternal race/ethnicity, age at the birth of child, parity, prepregnancy weight, smoking status, and weight gain during pregnancy. Single and multiple race groups were captured in the EHR and classified as Asian, Asian + Pacific Islander, Asian + white, or white. We calculated prepregnancy BMI using any weight measurement up to 1 year prior to date of pregnancy diagnosis; if more than one weight was available, we used the weight closest to the pregnancy index date. We divided BMI into four categories (underweight, normal weight, overweight, and obese) for both gBMI and aBMI indexes.

We calculated GWG using the difference between prepregnancy BMI and weight at the start of pregnancy. We allowed a window of 60 days after pregnancy diagnosis and 1 month prior to delivery to capture pregnancy weight at the start and end of pregnancy, respectively. GWG was classified into lower (“lGWG”), met (“mGWG”), and exceed (“eGWG”) GWG per WHO recommendations. Maternal age (years), smoking at any point during pregnancy (smoker vs. nonsmoker) and parity (no previous live births, one or more live births) and race/ethnicity were also derived from patients’ EHRs.

This study followed the “Strengthening the Reporting of Observational Studies in Epidemiology (STROBE) Statement: Guidelines for Reporting Observational Studies” and SAMPL Guidelines in reporting statistics. This study was exempt from Kaiser Permanente Hawaii Institutional Review Board approval because data were de-identified.

### Statistical Analysis

We calculated and summarized descriptive statistics, including means, standard deviations, and proportions (Table 1). Maternal age and BMI were treated as continuous variables; all other variables were treated as categorical. BMI was also treated as categorical when classified into the gBMI and aBMI groups as described above. The association between racial and ethnic group and each of the categorical and continuous variables was tested with chi-square and ANOVA respectively. People with data missing for a specific variable were excluded from the analyses that included that variable. Statistical analyses were conducted using SAS 9.4 (SAS Institute, Cary, NC). All reported statistical tests are two-sided; p-values < 0.05 were considered statistically significant. To allow the reader to interpret p-values more easily, we did not adjust for multiple comparisons.

**Table 1:** Characteristics of study population (n = 8886)

	Asian (n = 3623)	Asian + Pacific Islander (n = 2195)	Asian + white n = 732)	White (n = 2336)	p value^a^
Age (years), mean ± SD	31.9 ± 5.5	28.8 ± 5.7	30.7 ± 5.7	32.1 ± 5.3	< .0001
BMI (kg/m^2^), mean ± SD	24.8 ± 5.0	28.7 ± 6.8	26.0 ± 5.9	25.2 ± 5.7	< .0001
Gestational weight gain (lbs) per gBMI^b^ classification, mean ± SD					
Underweight	27.9 ± 9.1	34.6 ± 12.6	27.6 ± 11.5	31.43 ± 12.4	0.0017
Normal weight	28.1 ± 11.4	32.4 ± 11.8	31.1 ± 12.7	32.6 ± 12.3	< .0001
Overweight	25.1 ± 14.1	29.2 ± 14.6	28.2 ± 14.7	31.6 ± 14.0	< .0001
Obese	21.6 ± 20.8	24.3 ± 18.0	20.0 ± 15.8	22.9 ± 18.0	0.0141
Parity, n (%)					
0	1247 (34.4%)	535 (24.4%)	221 (30.2%)	930 (39.8%)	
≥ 1	2355 (65.0%)	1647 (75.0%)	509 (69.5%)	1393 (59.6%)	< .0001
Missing^c^	21 (0.6%)	13 (0.6%)	2 (0.3%)	13 (0.6%)	
Smoking, n (%)					< .0001
Yes	217 (6.0%)	325 (14.8%)	81 (11.1%)	152 (6.5%)	
No	3394 (93.7%)	1864 (84.9%)	647 (88.4%)	2178 (93.2%)	
Missing^c^	12 (0.3%)	6 (0.3%)	4 (0.5%)	6 (0.3%)	

^a^p-values based on ANOVA for age, BMI and gestational weight gain, and on chi-square for parity and smoking

^b^gBMI: WHO BMI cutoffs for the general population (underweight: < 18.5 kg/m^2^, normal weight: 18.5–24.9 kg/m^2^, overweight: 25–29.9 kg/m^2^, obese: ≥ 30 kg/m^2^)

^c^Excluded from statistical comparison

The cutoff for the underweight category remained the same for both gBMI and aBMI, and all women who were classified as overweight according to gBMI were reclassified as obese. Women assigned to the obese category under aBMI are therefore women in both the overweight and obese gBMI categories. Hence, racial and ethnic differences were analyzed only among normal weight and overweight women. Logistic regression, adjusting for a priori confounders (maternal age, parity, and smoking status) was used to estimate the odds of being reclassified.

Pregnancy weight gain for each was divided into three groups: lower than recommended GWG (lGWG), met recommended GWG (mGWG) and exceeded than recommended GWG (eGWG). We used the Cochran–Mantel–Haenszel test to check for linear associations between race/ethnicity and GWG across prepregnancy gBMI and aBMI categories.

## Results

### Population Characteristics

Among 11,684 women who had singleton live birth, 2798 (24%) were excluded due to missing prepregnancy and/or weight at 1 month prior to deliver. Women included in our analyses were comparable to women who were excluded in terms of mean age and smoking status. However, the women included were more likely to be multiparous because we selected the most recent pregnancy for women with multiple pregnancies.

A total of 8886 women (3623 Asian, 2195 Asian + Pacific Islander, 732 Asian + white, and 2336 white) met study criteria and were included in analyses (Table 1). Age was significantly different between race/ethnic groups (p < .0001), where Asian + Pacific Islander and white women had the lowest and highest mean ages, respectively. Prepregnancy BMI was also significantly different between race/ethnic groups (p < .0001): Asian women had the lowest mean prepregnancy BMI and were the only group with mean BMI within the normal gBMI category. The mean BMI of all other groups was in the overweight category, and Asian + Pacific Islander women had the highest mean BMI. GWG within gBMI categories varied across racial/ethnic groups. In the underweight category, Asian + Pacific Islander women gained the most weight, and Asian + white women gained the least. White women in the normal gBMI category gained the most weight, while Asian women gained the least. The same was seen in women in the overweight gBMI category. Among women classified by gBMI as obese, Asian + Pacific Islander women had the highest GWG, and Asian + white women had the lowest. Across all racial/ethnic groups, most women had given birth to at least one infant prior, although parity was significantly different across all racial/ethnic groups (p < .0001). The Asian + Pacific Islander group had the largest percentage of women who had previously given birth; white women had the smallest percentage. The majority of women did not smoke, although racial/ethnic differences were observed (p < .0001). The largest group of smokers were Asian + Pacific Islander women, while Asian women formed the smallest group.

### BMI Category Misclassification and Redistribution

A sizable number of women were reclassified into higher aBMI categories. Asian, Asian + Pacific Islander, and Asian + white women (17%, 11.8%, and 14.6%, respectively) were reclassified from normal gBMI to overweight aBMI (Table 2). Under aBMI, all women in the overweight gBMI category were reclassified into the obese category. The total percent of women who were reclassified from a gBMI category to a higher aBMI category was 42.1%, 40.2%, and 41.4% of Asian women, Asian + Pacific Islander women, and Asian + white women, respectively. After controlling for maternal age, parity, and smoking status, Asian + Pacific Islander women had the highest odds (vs. Asian) of being reclassified (OR 1.80, 95% CI 1.56, 2.07), as shown in Table 3.

**Table 2:** Prevalence of Reclassification from gBMI to aBMI by racial group

	Remained in normal BMI	Reclassified from gBMI normal category to overweight aBMI category	Reclassified from gBMI overweight category to obese aBMI category	Total reclassified from gBMI to higher aBMI category
Asian (n = 3623)	1412 (39.0%)	615 (17.0%)	909 (25.1%)	1524 (42.1%)
Asian + Pacific Islander (n = 2195)	440 (20.0%)	259 (11.8%)	623 (28.4%)	882 (40.2%)
Asian + white (n = 732)	254 (34.7%)	107 (14.6%)	196 (26.8%)	303 (41.4%)

*gBMI* WHO BMI categories based on the general population, *aBMI* WHO Asian-specific BMI categories

**Table 3:** Adjusted odds of being reclassified from gBMI category to a higher aBMI category among Asian and mixed-Asian (n = 4,788)

Effect	Adjusted odds ratio	(95% confidence limits)
Race/ethnicity		
Asian	**–**	
Asian + Pacific Islander	1.80*	(1.56, 2.07)
Asian + white	1.09	(0.91,1.31)

Odds ratios are based on multiple logistic regression and adjusted for maternal age, parity and smoking status

*p < .0001

*gBMI* WHO BMI categories based on the general population, *aBMI* WHO Asian-specific BMI categories

**Fig. 1:**
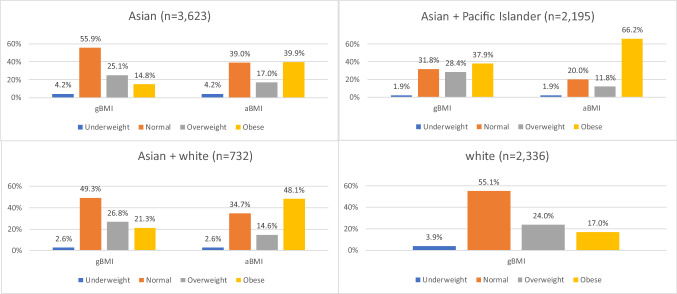
gBMI and aBMI distributions by race/ethnicity. gBMI: WHO BMI (underweight: < 18.5 kg/m^2^; normal weight: 18.5–24.9 kg/m^2^; overweight: 25–29.9 kg/m^2^; obese: > 30 kg/m^2^). aBMI: WHO Asian-specific cutoffs (underweight: < 18.5 kg/m^2^; normal weight: 18.5–22.9 kg/m^2^; overweight: 23–24.9 kg/m^2^; obese: ≥ 25 kg/m^2^)

Figure 1 shows BMI category distributions of by race/ethnicity according to gBMI and aBMI. When aBMI was applied, normal weight women were no longer the largest BMI group in Asian and Asian + white women. Instead, women in the obese category became the largest BMI group. Among Asian + Pacific Islander, women categorized as obese remained the largest group. Within the aBMI classification, across all ethnic groups, more women were in the overweight and obese categories combined than normal weight women.

### Racial/Ethnic Differences in Meeting Weight Gain Guidelines

**Fig. 2:**
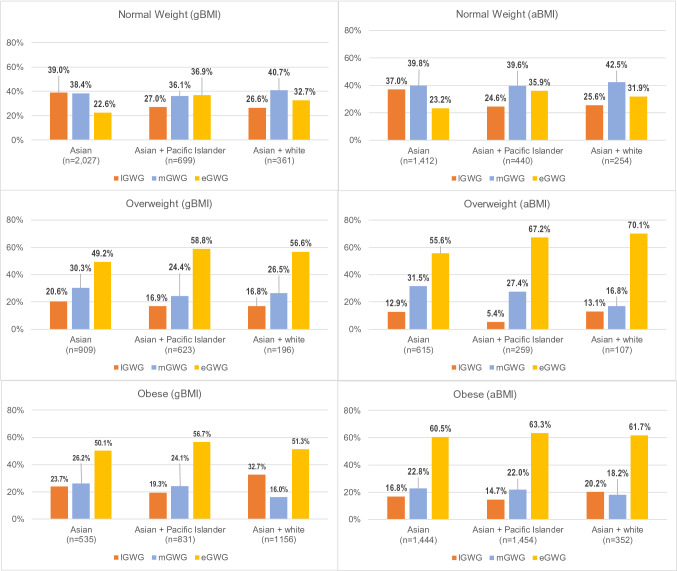
Racial/ethnic differences in meeting recommended gestational weight gain. gBMI: WHO BMI (underweight: < 18.5 kg/m^2^; normal weight: 18.5–24.9 kg/m^2^; overweight: 25–29.9 kg/m^2^; obese: > 30 kg/m^2^). aBMI: WHO Asian-specific cutoffs (underweight: < 18.5 kg/m^2^; normal weight: 18.5–22.9 kg/m^2^; overweight: 23–24.9 kg/m^2^; obese: ≥ 25 kg/m^2^). lGWG indicates those whose gestational weight gain was less than WHO recommendation for BMI category: normal BMI category < 25 lbs; overweight BMI category < 15 lbs; obese BMI category < 11 lbs. mGWG indicates those whose gestational weight gain was within WHO recommendation for BMI category: normal BMI between 25 and 35 lbs; overweight BMI between 15 and 25 lbs; overweight BMI between 11 and 20 lbs. eGWG indicates those whose gestational weight gain was greater than WHO recommendation for BMI category: normal BMI ≥ 35 lbs; obese BMI > 25 lbs; obese BMI > 20 lbs

We found racial/ethnic differences in the eGWG group among normal weight and overweight women. Within the normal BMI category, the Asian + Pacific Islander had the largest eGWG group, followed by Asian + white women; this finding was observed when using gBMI (p < .0001) and aBMI (p = .0029). Racial/ethnic differences were also observed among the overweight women (gBMI p = .0005; aBMI p = .006). Using gBMI cutoffs, Asian + Pacific Islander had the largest eGWG group. However, when aBMI was applied, Asian + white women had the largest eGWG group (Fig. 2).

Among overweight women, eGWG group was the largest weight-gain group under both gBMI and aBMI. In changing from gBMI to aBMI, the eGWG group increased—the largest increase was seen in overweight Asian + white women.

## Discussion

To the best of our knowledge, this study is the first to report on the prevalence of overweight and obese prepregnancy BMI and eGWG in mixed-race Asian women. We showed that the application of aBMI cutoffs increased an already high number of women who were considered overweight or obese at the start of their pregnancy, especially among Asian + Pacific Islander women. While the prevalence of reclassification was similar among the three groups, Asian + Pacific Islander women were nearly twice as likely to be reclassified, after adjusting for age, parity, and smoking. Finally, we saw ethnic variability in GWG, where mixed-race Asian women exceeded recommended GWG more than Asian women.

Our findings were similar to results published by Gao et al. (2020), the only existing study that compared the prevalence of overweight and obese prepregnancy BMI among Asian women using both gBMI and aBMI cutoffs. Utilizing data on more than 1 million Asian women from the National Vital Statistics System, the study reported that 17.4% of women were reclassified from normal BMI to overweight BMI when aBMI was applied, which is similar to results from our study (11.8–17%). As Gao et al. excluded all mixed-race and Pacific Islander women, the closest comparison group in our study would be the Asian group, in which 17% of the women were reclassified from normal gBMI.

While we were unable to compare reclassification from overweight to obese between the studies due to different cutoffs for obesity, the combined percentage of women in the overweight and obese categories was comparable (40.2–42.1% of our study population vs. 46.8% in Gao et al.). Of note, reclassification using aBMI resulted in the obese category becoming the largest group among all race/ethnic group. The high prevalence of overweight and obesity among Asian women in our study is only slightly lower than the prevalence of prepregnancy overweight and obesity found among a national sample of U.S. women (Deputy et al., 2018).

Mixed-race Asian women had a higher prevalence of exceeding recommended GWG than Asian women. This was especially true among the overweight gBMI and aBMI groups, where most women in each group exceeded GWG guidelines, with mixed-race Asian women leading in prevalence. Although we do not know what the optimal GWG for Asian women should be, various studies outside of the U.S. have demonstrated that Asian women who gained less weight than recommended by IOM had better pregnancy outcomes. Studies from Asian countries using aBMI cutoffs have found that GWG that is less than IOM recommendations is associated with reduced risks of gestational hypertension (Choi et al., 2017; Morisaki et al., 2017; Park et al., 2011), C-section (Choi et al., 2017; Ee et al., 2014; Park et al., 2011) and large for gestational age (Chen et al., 2015; Choi et al., 2017; Ee et al., 2014; Morisaki et al., 2017; Ota et al., 2011). The disparities between Asian and mixed-race Asian groups in the overweight and obese BMI classifications warrant further research. Acculturation is associated with GWG Understanding modifiable factors that contribute to these differences in prepregnancy BMI and GWG will inform interventions for better maternal and child outcomes.

### Strengths and Limitations

Interpretation of our study results should be done in light of its limitations. We were not able to break down specific Asian and mixed-race Asian groups such as Chinese, Japanese, Hawaiian and Samoan because our sample size was not large enough. Our study population was derived from Asian women in Hawaii only and thus not generalizable to the rest of the country. Ours was a secondary analysis of existing data, and we did not have the opportunity to collect additional information such as psychosocial and other social determinants of health. Immigrant women in the United States have lower prepregnancy obesity than U.S.-born women (Singh & DiBari, 2019), and we were not able to adjust for nativity in our analyses. In addition, women with lower income and education have higher BMIs (Singh & DiBari, 2019), which might explain differences in the number of women who were reclassified. Best practice alerts notify clinicians of the need to recommend a range of GWG to patients; however, we cannot confirm that all women in our study were informed about their recommended GWG. Women included in our analyses were more likely to be multiparous. Nulliparous women tend to have fewer and less frequent health care utilization than multiparous women (Lau et al., 2014) (Kurata et al., 2020; Lau et al., 2014) and were therefore less likely to have had a pre-pregnancy BMI measured in the study time period. Studies indicate that multiparous women start their pregnancy at a higher weight (Ziauddeen et al., 2019) and gain more weight during pregnancy (Lan-Pidhainy et al., 2013) suggesting that the pre-pregnancy BMI and GWG in our study may be on the higher end and not generalizable to all pregnant women.

Strengths of our study include detailed racial/ethnic data that allowed us to delineate Asian, Asian + Pacific Islander, and Asian + white groups. Another strength of our study was the use of EHR records for GWG data, which were measured by clinicians rather than self-reported.

### Conclusions

In light of the many studies demonstrating the association of high prepregnancy BMI with adverse outcomes (Choi et al., 2011; Soltani et al., 2017; Sun et al., 2020), we find it worrisome that application of an aBMI cutoff resulted in more women being classified as overweight/obese than normal weight across all Asian groups in our study. The possibility exists that a large proportion of these women were given weight gain goals that might have been higher than optimal in a population where a significant number of women already exceed GWG recommendations. Including aBMI to estimate GWG goals, along with individualized prenatal care for Asian women, may be the first step toward better pregnancy outcomes.

## Data Availability

Please contact Dr. Yihe Daida at Yihe.G.Daida@kp.org.
